# ALK-Rearranged NSCLC With Concomitant HER2-Mutant Breast Cancer Patient Treated With Alectinib, Trastuzumab, and Pertuzumab: A Case Report

**DOI:** 10.7759/cureus.36711

**Published:** 2023-03-26

**Authors:** Hirokazu Takayasu, Yuki Kata, Yukiko Otsu, Satoshi Inoue, Takeshi Kaneko

**Affiliations:** 1 Respiratory Medicine, Yamato Municipal Hospital, Yamato, JPN; 2 Pulmonology, Yokohama City University Graduate School of Medicine, Yokohama, JPN

**Keywords:** breast cancer alk-rearranged, pertuzumab, trastsuzumab, alectinib, her2-mutant, non-small cell lung cancer

## Abstract

Multiple cancers are a common occurrence, and the choice of treatment can be a challenging decision. The current case report describes a 71-year-old woman with overlapping anaplastic lymphoma kinase (ALK)-rearranged lung adenocarcinoma and HER2-mutant breast cancer, who achieved improvement with concurrent use of the molecularly targeted agents Alectinib, Trastuzumab, and Pertuzumab.

A 71-year-old woman was diagnosed with lung adenocarcinoma and brain metastases, and invasive ductal carcinoma of the right breast, HER2-mutant type. In March 2021, a biopsy confirmed the presence of the ALK fusion gene in lung cancer. In April 2021, he started Alectinib and showed shrinkage of lung cancer; in December 2021, a metastatic liver tumor was found, and a liver biopsy diagnosed liver metastasis of breast cancer. Therefore, Alectinib was discontinued in February 2022, and Trastuzumab, Pertuzumab, and Docetaxel were started as chemotherapy for breast cancer. She continued treatment with Trastuzumab and Pertuzumab, but in July 2022, she developed an increase in lung cancer. Her metastatic liver tumor continued shrinking, and she was started on Trastuzumab, Pertuzumab, and Alectinib. After six months of treatment, the patient showed a sustained reduction in both lung cancer, breast cancer, and brain metastases with no adverse events. ALK rearrangement lung cancer often develops in young women, and similarly, breast cancer often develops in women. Therefore, those cancers may occur simultaneously. In such cases, the choice of treatment can be difficult, as both cancers require different approaches. Alectinib has been shown to have a high response rate and prolonged progression-free survival in ALK-rearranged non-small cell lung cancer (NSCLC). Trastuzumab and Pertuzumab are commonly used for the treatment of HER2-mutant breast cancer and have been shown to significantly improve progression-free survival and overall survival. This case report provides evidence that the concurrent use of Alectinib, Trastuzumab, and Pertuzumab can be an effective treatment for patients with overlapping ALK-rearranged NSCLC and HER2-mutant breast cancer. It is important to consider concurrent treatment in patients with multiple cancers to optimize treatment outcomes and improve quality of life. However, further studies are needed to establish the safety and efficacy of this combination of drugs for the treatment of overlapping cancers.

## Introduction

Lung cancer, particularly non-small cell lung cancer (NSCLC), remains a daunting challenge for oncologists, with a multitude of genetic alterations driving tumorigenesis and progression. The heterogeneity of NSCLC requires the adoption of precision medicine approaches, tailored to the specific genetic and molecular aberrations driving each individual case. One such genetic alteration that has garnered substantial attention is the Anaplastic Lymphoma Kinase (ALK) gene rearrangement, which is estimated to occur in approximately 3%-7% of NSCLC cases and is associated with a distinctive clinicopathological phenotype [1].

The identification of the ALK gene rearrangement has led to the development of tyrosine kinase inhibitors (TKIs), such as Crizotinib and Alectinib, which have revolutionized the treatment of ALK-rearranged NSCLC. These TKIs target the oncogenic ALK fusion protein and have shown remarkable efficacy in managing this subtype of NSCLC, with a high proportion of patients experiencing objective response and durable disease control [2,3]. However, despite the initial success of TKIs, acquired resistance remains a significant obstacle, limiting the durability of response and highlighting the need for a deeper understanding of the underlying resistance mechanisms. In this study, we present a case of a 71-year-old woman with ALK-rearranged NSCLC who also developed Human epidermal receptor 2 (HER2)-mutant breast cancer. The occurrence of ALK-rearranged NSCLC and HER2-positive breast cancer is a rare and challenging clinical scenario, and there is limited information on the best approach for its management. Typically, Trastuzumab and Pertuzumab, which are anti-HER2 therapies, are used together for recurrent cases of HER2-positive breast cancer after surgery [4]. However, the management of patients with multiple malignancies requires a personalized, multi-disciplinary approach, taking into consideration the specific molecular and genetic aberrations driving each tumor.

Our case report highlights the use of Alectinib, a TKI, in combination with Trastuzumab and Pertuzumab for the concurrent treatment of ALK-rearranged NSCLC and HER2-mutant breast cancer. We have demonstrated that the combination of molecularly targeted agents with different mechanisms of action in patients with two different types of advanced cancer has succeeded in each of these patients. This report underscores the importance of a personalized therapeutic approach in managing patients with multiple malignancies and the need for further research on the optimal combination of therapies in such cases. Our findings may contribute to developing novel therapeutic strategies to improve the prognosis of patients with coexisting ALK-rearranged NSCLC and HER2-mutant breast cancer.

## Case presentation

**Figure 1:**
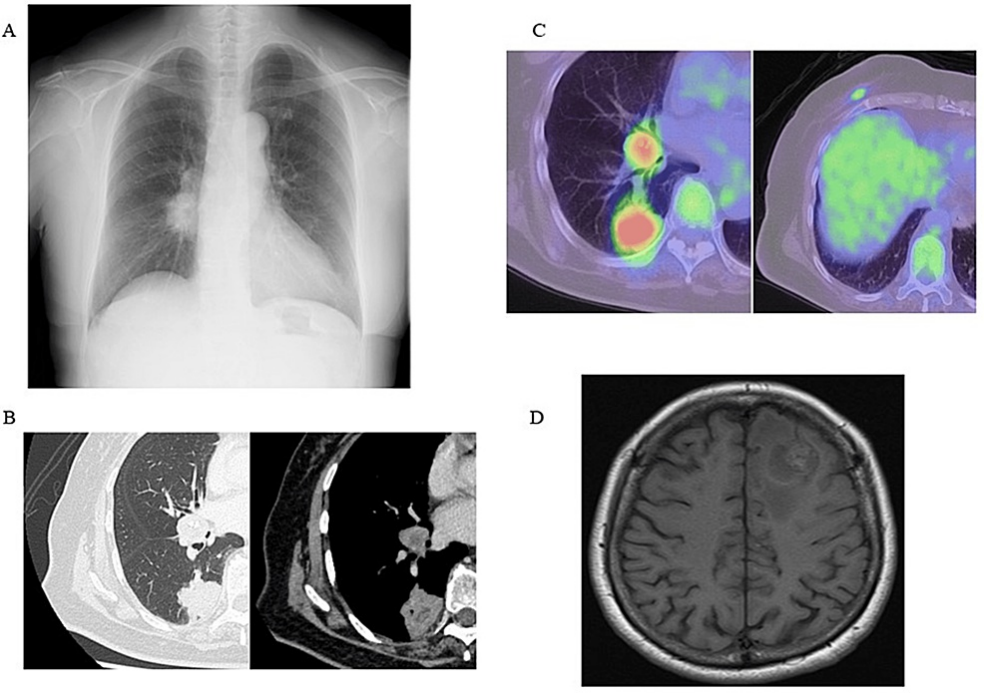
Chest x-ray, computed tomography (CT) findings, positron emission tomography (PET), and magnetic resonance imaging (MRI) of the head before starting Alectinib therapy (A) Chest x-ray shows a mass in the right hilar region. (B) CT scan of the chest shows lung cancer in the right lower lobe and lymph node metastasis in the hilar region. (C) PET scan shows FDG accumulation in right lower lobe lung cancer, lymph node metastasis, and right breast cancer. (D) Head MRI shows a tumor in the left frontal lobe.

A 71-year-old woman was diagnosed with right lower lobe lung adenocarcinoma with brain metastasis and HER2-mutant type right invasive ductal carcinoma of the breast. The patient was a non-smoker and in good health, presented to the hospital in January 2021 with a right breast mass. A 25-mm tumor was found in the right mammary gland, and a breast biopsy was performed. The tumor was diagnosed as thyroid transcription factor-1 (TTF-1) negative, HER2-mutant, estrogen receptor (ER)-negative, progesterone receptor (PgR)-negative invasive ductal carcinoma of the breast. In addition, a chest x-ray and computed tomography (CT) scan showed a mass in the lower lobe of the right lung (Figures 1A, 1B). Positron emission tomography (PET) scan showed a 60-mm tumor in the lower lobe of the right lung, 18F-fluorodeoxyglucose (FDG) accumulation in the right hilar region, sub-tracheal bifurcation, and paratracheal lymph nodes and the standardized uptake value (SUV) max of the right lung tumor was 8.2 (Figure 1C). Contrast-enhanced magnetic resonance imaging (MRI) of the head showed a tumor in the brain (Figure 1D). A lung biopsy was performed in March 2021, and a diagnosis of ALK fusion gene mutant lung adenocarcinoma was made by high-sensitivity immunohistochemistry (IHC) and fluorescence in situ hybridization (FISH). Based on these results, the patient was determined to have concurrent right lower lobe lung adenocarcinoma (cT2aN1M1c, BRA, UICC 7th edition) and right breast duct cancer (cT1N0M0, UICC 7th edition). The brain tumor was determined to be a metastasis of lung cancer as there was no evidence of axillary lymph node metastasis of breast cancer.

**Figure 2:**
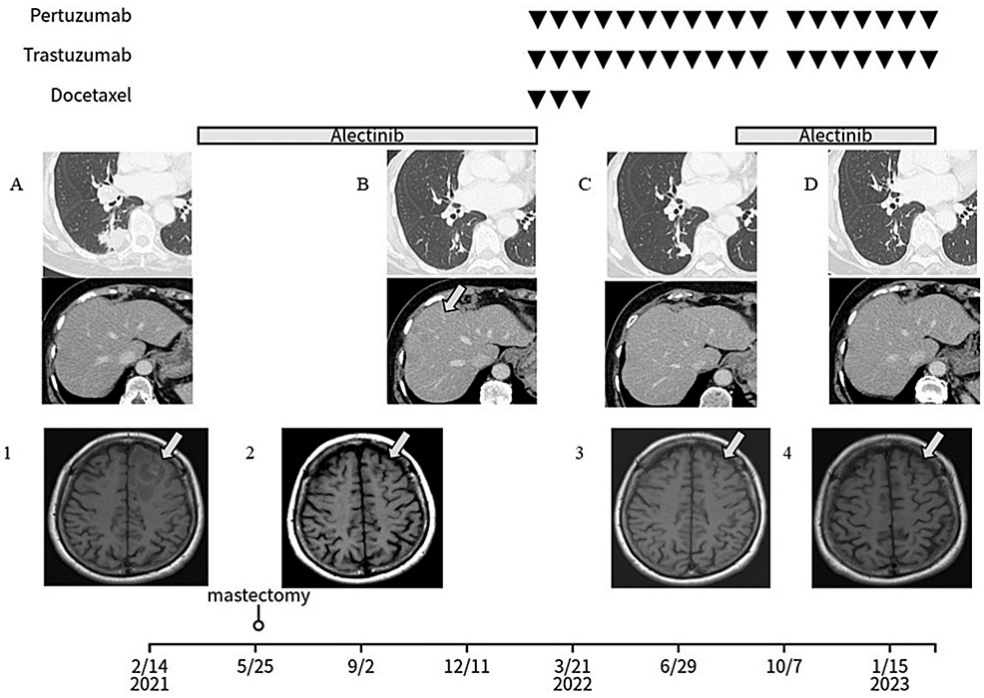
Clinical course Contrast-enhanced CT (A-D): lung cancer (upper row), metastatic liver tumor (liver metastasis of breast cancer) (lower row). (A) February 2021, (B) December 2021, (C) July 2022, (D) February 2023. Contrast-enhanced MRI of the head (1-4) (1) February 2021, (2) October 2021, (3) August 2022, (4) November 2022.

The first dose of Alectinib 600 mg/day was started in April 2021. The patient tolerated the drug well, and a right mastectomy was performed in May 2021 while the medication was continued. Postoperatively, Alectinib was continued, and a head MRI scan in October 2021 (Figure 2.2) showed shrinkage of brain metastases. The patient's general condition was good, but a CT scan in December 2021 revealed a new metastatic liver tumor (Figure 2B). Liver biopsy was performed because it was unclear whether the primary of this metastatic liver tumor was lung or breast cancer. The obtained tissue showed infiltration and proliferation of malignant cells identical to those of breast cancer, and immunostaining was negative for TTF-1, leading to a diagnosis of metastatic liver tumor derived from breast cancer. In February 2022, Alectinib was discontinued, and combination therapy with Pertuzumab, Trastuzumab, and Docetaxel was initiated; after three courses of treatment, the patient was switched to a combination of Pertuzumab and Trastuzumab in May 2022 and completed eight courses in July 2022. CT in July 2022 showed liver metastases had shrunk, but lung cancer had increased (Figure 2C), and a subsequent head MRI showed a slightly enlarged brain tumor (Figures 2.2, 2.3). Treatment for lung cancer needed to be resumed, and Alectinib, Trastuzumab, and Pertuzumab were started simultaneously in August 2022. The schedule was delayed only once due to a bout of bronchiolitis, but the combination therapy was continued according to the protocol. A head MRI in November 2022 showed no increase in brain tumor (Figures 2.2-2.4), and a CT in February 2023 showed shrinkage of lung cancer and liver metastases (Figure 2D). Six months after the initiation of combination therapy, the patient is asymptomatic, no adverse events have been observed, and the treatment response is good. Tumor markers frequently used in lung and breast cancer, such as CEA, CA19-9, and CA15-3 were consistently below baseline values during this patient. No other biomarkers reflecting disease status were detected in biochemical laboratory tests. 

## Discussion

ALK fusion gene expression is found in approximately 5% of NSCLC. In these cells, the ALK tyrosine kinase is thought to be persistently activated, leading to uncontrolled cell growth and tumorigenesis [1,5]. Alectinib targets the ALK tyrosine kinase to inhibit tumor growth and exert its antitumor effect [6]. On the other hand, HER2 is a cell surface-expressed protein implicated in disease pathogenesis in approximately 25% of breast cancer patients. HER2 is a protein expressed on the cell surface and implicated in disease pathogenesis in approximately 25% of breast cancer patients. Trastuzumab and Pertuzumab, molecular targets of HER2, are vital in treating HER2-mutant breast cancer and improving progression-free survival and overall survival [7,8].

Drug therapy for advanced cancer can be broadly classified into cytotoxic chemotherapy, molecularly targeted drugs, and immune checkpoint inhibitors. Cytotoxic chemotherapy is not specific to patients or their organs, and its efficacy cannot be determined without actual administration since the therapeutic effect cannot be predicted. In addition, they are highly injurious to normal cells, resulting in myelosuppression, alopecia, and gastrointestinal disorders. In contrast, molecular-targeted therapies can predict and limit the therapeutic effect and patients who can be expected to respond to the therapy in advance by examining specific cancer genes and proteins, thereby enabling highly selective treatment with minimal effect on normal cells. However, it may cause characteristic side effects different from those of conventional anticancer drugs. For example, drug-induced lung injury requires careful attention. Immune checkpoint inhibitors activate autologous T cells to eliminate cancer cells indirectly. As a result, they may be effective even in advanced cancers that no longer respond to Cytotoxic chemotherapy, and they tend to have a long-lasting therapeutic effect in some patients. On the other hand, high medical costs and immune-related adverse events are problematic. Each of the above therapeutic agents has advantages and disadvantages, and the use of appropriate molecular targeted agents in patients with advanced cancer has been shown to prolong survival [9].

However, the usefulness of the simultaneous use of multiple molecular-targeted agents for different cancers continues to be debated due to limited information. Some studies suggest that there may be NSCLC patients with both EGFR mutations and ALK- rearrangements and that combination treatment with an EGFR inhibitor and an ALK inhibitor may be effective [10]. Alectinib is effective in advanced ALK-rearranged NSCLC, but the response is limited due to the development of drug resistance [11]. HER2 gene amplification has been reported as a resistance mechanism, making combined drug therapy a promising strategy. Trastuzumab Deruxtecan has been found to have sustained anticancer activity and safety in patients with HER2-mutant NSCLC [12]. Patients with EGFR-mutant lung cancer are more likely to present with independent overlapping cancers, making the concurrent use of different molecularly targeted agents more likely [13]. This case report describes Alectinib, Trastuzumab, and Pertuzumab to treat lung and breast cancer, resulting in sustained shrinkage of the tumors and multiple brain metastases. This suggests that the concurrent administration of multiple molecularly targeted agents is a feasible and safe strategy without compromising efficacy.

The strengths of this case study include providing insight into the efficacy of treating overlapping cancers with concurrent administration of multiple molecularly targeted agents, maintaining the patient's quality of life without significant adverse events, and being an example of the effectiveness of simultaneous administration of different regimens of anticancer drugs. Limitations of this case study include the fact that it evaluated a limited number of drugs, and it is yet to be known whether the study can be applied to other frequently used molecular-targeted drugs. Further confirmation of long-term efficacy and side effects is needed because the treatment period was six months. Nevertheless, the results of this case study support the use of multiple molecular-targeted agents simultaneously in the treatment of overlapping cancers and suggest that this may be a promising therapeutic strategy.

## Conclusions

In this study, we have successfully treated a patient with combined lung and breast cancer using different molecularly targeted agents. It highlights the feasibility and potential benefits of using molecularly targeted agents in managing patients with overlapping cancers. The use of these agents in the treatment of advanced cancer can potentially improve patient outcomes. It should be an essential consideration in the management of these patients. Further studies are needed to establish these agents' efficacy and safety in treating patients with multiple cancers.
